# Development of A Guideline for Reporting Mediation Analyses (AGReMA)

**DOI:** 10.1186/s12874-020-0915-5

**Published:** 2020-02-03

**Authors:** Aidan G. Cashin, James H. McAuley, Sarah E. Lamb, Sally Hopewell, Steven J. Kamper, Christopher M. Williams, Nicholas Henschke, Hopin Lee

**Affiliations:** 1 Neuroscience Research Australia, Sydney, Australia; 2 Prince of Wales Clinical School, Faculty of Medicine, University of New South Wales, Sydney, Australia; 3 School of Medical Sciences, Faculty of Medicine, University of New South Wales, Sydney, Australia; 4 Centre for Statistics in Medicine, Nuffield Department of Orthopaedics Rheumatology and Musculoskeletal Sciences, University of Oxford, Oxford, UK; 5 Institute of Health Research, University of Exeter Medical School, Exeter, UK; 6 Institute for Musculoskeletal Health, Faculty of Medicine and Health, University of Sydney, Sydney, Australia; 7 Centre for Pain, Health and Lifestyle, New Lambton Heights, Australia; 8 School of Medicine and Public Health, University of Newcastle, Newcastle, Australia; 9 School of Public Health, University of Sydney, Sydney, Australia

**Keywords:** Mediation analysis, Mechanisms, Reporting guideline

## Abstract

**Background:**

There are a growing number of studies using mediation analysis to understand the mechanisms of health interventions and exposures. Recent work has shown that the reporting of these studies is heterogenous and incomplete. This problem stifles clinical application, reproducibility, and evidence synthesis. This paper describes the processes and methods that will be used to develop a guideline for reporting studies of mediation analyses (AGReMA).

**Methods/design:**

AGReMA will be developed over five overlapping stages. Stage one will comprise a systematic review to examine relevant evidence on the quality of reporting in published studies that use mediation analysis. In the second stage we will consult a group of methodologists and applied researchers by using a Delphi process to identify items that should be considered for inclusion in AGReMA. The third stage will involve a consensus meeting to consolidate and prioritise key items to be included in AGReMA. The fourth stage will involve the production of AGReMA and an accompanying explanation and elaboration document. In the final stage we will disseminate the AGReMA statement via journals, conferences, and professional meetings across multiple disciplines.

**Discussion:**

The development and implementation of AGReMA will improve the standardization, transparency, and completeness in the reporting of studies that use mediation analysis to understand the mechanisms of health interventions and exposures.

## Background

The effects of exposures and health interventions are presumed to work via biological or psychosocial mechanisms. In recent years, epidemiologists and clinical trialists have used mediation analysis to understand the causal mechanisms by which exposures and interventions exert their effects on health outcomes [1–3]. Mediation analysis is a quantitative method for evaluating causal mechanisms where the effect of interest is partitioned into “indirect effects” that work through the mechanism(s) of interest, and a “direct effect” that works through all other unspecified mechanisms [4]. The use of mediation analysis to understand the mechanisms of health interventions has been advocated by the US National Institute of Health (NIH), UK National Institute for Health Research (NIHR), and UK Medical Research Council (MRC) [1, 2].

While the use of mediation analysis has become increasingly common in recent years, there is growing recognition that the reporting of studies that use mediation analysis to investigate causal mechanisms of healthcare interventions is heterogenous and often incomplete [1, 5–11]. A recent overview of reviews across 11 health care fields and 26 healthcare conditions showed that mediation studies often did not report effect sizes and precision estimates (14/54, 26%), the theoretical rationale for the mechanism being tested (7/54, 13%), and essential details of the analytical techniques (4/54, 7%) [1]. Reviews of primary mediation studies also show that the reporting of effect estimates and assumptions are varied across the literature [5] and that most mediation analyses of randomised trials did not report sample size calculations [11]. These limitations stifle clinical application, reproducibility, and evidence synthesis.

Reporting guidelines can improve the transparency, consistency, completeness and reproducibility of research reports [12–14]. Existing reporting guidelines such as the Consolidated Standards of Reporting Trials (CONSORT) [15], Strengthening the Reporting of Observational Studies in Epidemiology (STROBE) [16] and their extensions are not directly applicable to mediation analysis, and there is no specific guidance for the reporting of studies that use mediation analysis. Considering that mediation analysis is commonly applied to both randomised controlled trials and observational studies and sometimes reported separate to the original study, an extension of the CONSORT or STROBE would not be suitable and instead, a specific reporting guideline is required. A specific reporting guideline for mediation studies would guide researchers to transparently report a minimum set of items that would represent the methodology and findings, in particular, reflecting issues that may introduce or prevent bias [17].

To overcome the problem of suboptimal reporting of mediation studies, the US Berkeley Initiative for Transparency in the Social Sciences and the Center for Effective Global Action funded a project to develop a specific reporting guideline for mediation studies. This paper describes the processes and methods that will be used to develop AGReMA - A Guideline for Reporting Mediation Analyses.

## Methods/design

**Fig. 1:**
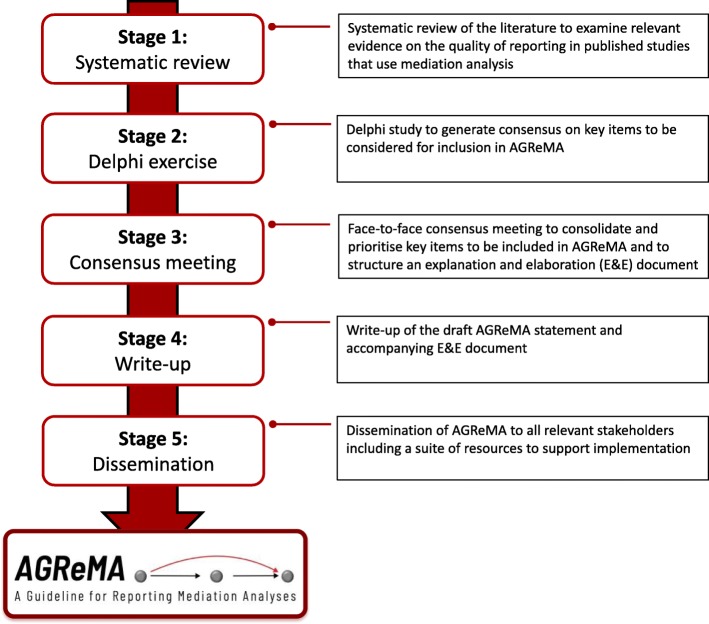
Workflow for the development of AGReMA: A Guideline for Reporting Mediation Analyses

AGReMA will be developed over five overlapping stages in accordance with the guidance for development of health research reporting guidelines [18] – Fig. 1

It is anticipated that the first two stages of the guideline development will be completed prior to the Consensus meeting scheduled to be held in Oxford late Spring 2020. The Dissemination of AGReMA is planned for early 2021.

### AGReMA working group

The AGReMA working group is made up of the project leads (HL, AC, JM) and five advisory members (SL, SH, SK, CW, NH). The group was established to collate expertise on the application of mediation analysis to clinical trials and observational studies, evidence synthesis, reporting guideline development, editorial experience and research implementation. The AGReMA working group developed the project protocol, secured funding and will be responsible for conducting each stage of the guideline development. In addition, the working group will help recruit key stakeholders for stages 2 and 3 and write the guideline documents along with relevant stakeholders.

### Stage 1 – systematic review

This study aims to systematically review the quality of reporting in published studies that use mediation analysis. Assessing the quality of reporting will provide important insights into the prevalence of potential sources of bias in studies that use mediation analysis and on reporting items to be considered for the eventual guideline [18]. Vo et al. 2019 reviewed the reporting quality of randomised trials that use mediation analysis [11]. We will systematically review the reporting quality of non-randomised observational studies that have used mediation analysis to investigate causal mechanisms. The protocol for this systematic review was registered on the 29th May 2019 with the International Prospective Register of Systematic Reviews (PROSPERO ID: CRD42019136348).

#### Databases and search terms

We will search EMBASE (OvidSP), MEDLINE (OvidSP) and PsycINFO (OvidSP) for non-randomised observational studies published in the previous two years that used mediation analysis. The search dates will be restricted to the most recent two years to focus on current reporting practices. We will use the following search terms: mediation analysis, causal mediation, structural equation modelling, product of coefficient, indirect effect, direct effect, mechanism, intermediate variables [19]. We will not restrict our search based on health condition, journal, or type of exposure/intervention; mediator(s) and outcome(s) investigated to ensure a representative sample of up to 50 studies across healthcare. Our sample size was informed by previous systematic reviews that investigated the reporting quality of epidemiological studies [20, 21].

#### Data screening and selection

After removing duplicates, we will randomly order the identified records and select a random sample of up to 50 studies. We will include non-randomised observational studies (cohort, case-control, cross-sectional and non-randomized controlled trials) that used mediation analysis to understand the mechanisms of any health intervention or exposure for individuals with any health condition, or at risk of developing any health condition. We will exclude reports of randomized controlled trials, non-randomised observational studies that have not used mediation analysis, articles for which full texts are unavailable and non-English language studies. We will not exclude studies based on their comparator group or the reported outcome. Two reviewers will independently apply the inclusion/exclusion criteria sequentially to the random sample until perfect agreement is achieved between reviewers. Following this, one reviewer will screen the remaining studies independently until 50 studies have been included. Disagreements between reviewers at this stage will be discussed and resolved by consensus.

#### Data extraction

A customised data extraction form will be developed and piloted tested using 10 studies. After piloting, a single reviewer will independently extract all data. A second reviewer will verify the data extraction for 10% of the included studies with discrepancies to be resolved through discussion. If the discrepancies total greater than 20% of possible data items, the second reviewer will verify all of the remaining data. First, we will extract descriptive information from each study including: authors; year of publication; journal; healthcare field; study design (cohort, case-control, cross-sectional and non-randomized controlled trials); publication type (primary or secondary publication using mediation analysis); aim of study; sample size; health condition; exposure/intervention; comparison group; outcomes measured; outcome measures; mediators investigated; mediators measures; time points measured. Secondly, we will extract specific information about the reporting quality of the methods and results of the mediation analysis. The assessment of reporting quality will be guided by the reporting of items identified from a scoping review of existing methodological and reporting guidance documents for mediation analyses, and from the findings of our recent overview of systematic reviews [1]. The assessment focuses on reporting items considered essential to appropriately interpret and reproduce a study that uses mediation analysis. These include the theoretical rationale and study design for testing the mechanism of interest, details of the analytic technique and the reporting of effects unique to mediation analyses such as indirect and direct effects. Disagreement during data extraction will be resolved through consensus and where necessary, by a third independent reviewer. Study data will be managed using the Research Electronic Data Capture (REDCap) tool [22].

#### Data synthesis

We will summarise the descriptive information using frequencies and percentages for categorical variables and mean and standard deviation or median and interquartile range for normal and non-normal distributed continuous variables respectively. We will report the number and proportion of studies which report each of the prespecified reporting items.

#### Outcomes of systematic review

The results of this review will provide evidence on the quality of reporting of non-randomised observational studies that use mediation analysis. These findings will be used alongside existing systematic reviews [5, 10, 11] to inform the Delphi study (stage 2), and the consensus meeting (stage 3) to decide on the core reporting items for AGReMA. The findings of this review will be submitted for publication.

### Stage 2 – Delphi study

The aim of the Delphi study is to seek expert agreement on a list of items that should be reported in a mediation study. The process includes consulting experts to (1) assess the level of agreement on an initial list of reporting items generated from previous reviews; (2) elicit additional items and refine the initial list; and (3) identify which items are considered most important in reporting mediation studies to inform the consensus meeting.

#### Ethics

Ethics approval has been obtained from the University of New South Wales Human Research Ethics Advisory Panel D: Biomedical, approval number HC16599.

#### Design

The Delphi technique is a structured method to achieve consensus among a panel of experts on a given question or topic [23]. This process will comprise of a series of questionnaires or ‘rounds’, where panellists independently and anonymously contribute and rank items. This process is repeated for 3 rounds, or until consensus is reached. Following each round, panellists will be provided with summary feedback to encourage the reassessment of judgements for subsequent rounds, assisting in transforming individual opinion into group consensus [23].

#### Selection of preliminary items

We will collate a preliminary list of items to be considered in round 1 of the Delphi based on results from the systematic review (stage 1) and existing methodological and reporting guidance documents for mediation analyses. In addition, we will examine items from the CONSORT and STROBE checklist [15, 16] to identify potential reporting items that could be adapted for mediation studies.

#### Participants

We will invite experts who represent key stakeholders, including methodologists, statisticians, systematic reviewers, journal editors, implementation scientists, applied researchers, psychologists and clinical researchers to be included in the Delphi panel. We will invite experts who have published original research papers involving mediation analysis or systematic reviews of mediation studies; methodological/statistical research papers on mediation analysis; or textbooks on mediation analysis. Unlike representative surveys, the Delphi method is a consensus exercise involving experts and does not require large sample sizes for statistical power. In accordance with Fitch et al. (2001), we will aim to include between 7 to 15 participants in the Delphi study [24].

#### Recruitment process

Potential panellists will be identified through a variety of sources, including an overview [1] and a scoping review of the literature, and through consultation with experts. Recruitment will be iterative, with the final list of potential panellists decided through consensus amongst the AGReMA working group.

#### Procedure

CLINVIVO (www.clinvivo.com), an independent company will co-ordinate the web-based Delphi study to limit biases from the AGReMA working group [25]. Each Delphi round will be open for 3 weeks. Reminder emails will be sent to non-completers every 7 days until they complete the round or until the round closes. We will also highlight the importance of completing all three rounds of the Delphi study and only invite panellists who completed the previous round to take part in subsequent rounds [26]. Panellist responses will be de-identified by CLINVIVO to maintain anonymity between panellists and from the AGReMA working group.

#### Round 1

A questionnaire will be sent via email to the panellists using CLINVIVO‘s bespoke electronic data capture program. The questionnaire will include a statement about the purpose of project, demographic questions and reporting items for consideration. Panellists will be asked to score the importance of each potential reporting item on a 9-point Likert scale (1,” not important”, to 9, “critically important”) and to describe their confidence in their ratings (1, “not confident”, to 9, “very confident”). Free text space will be provided at the end of each section to enable panellists to provide suggestions on wording and to allow panellists to suggest articles which could support item inclusion/exclusion. In addition, panellists will be asked to contribute additional items for consideration in subsequent rounds.

#### Round 2

Panellists who complete the first round will be sent a second-round questionnaire. This will include a summary of results from round one (mean scores and their standard deviations, median scores and inter-percentile ranges (IPR) (30th and 70th), histograms and Research ANd Development/University of California Los Angeles (RAND/UCLA) labels (see analysis below) of importance and agreement level), together with the panellist’s own score for each item. Newly nominated items and suggested re-wording of items from round 1 will also be presented. Panellists will be invited to re-score the importance of each item in the light of the aggregated panel medians. Panellists will be reminded that items scored ≤3 are considered not important and will be excluded and items scored ≥7 are considered critically important and will be included for consideration in the reporting guideline. Free text space will be provided at the end of each section to enable panellists to provide further suggestions on wording.

#### Round 3

Panellists who complete the second round will be sent a third-round questionnaire including a summary of results from round two (mean scores and their standard deviations, median scores and IPRs (30th and 70th), histograms and RAND/UCLA labels of importance and agreement level) for each item alongside the panellist’s own score. Panellists will be informed about the items that reached consensus for inclusion (median score ≥ 7) and exclusion (median score ≤ 3). Panellists will be asked to rate the remaining items for which consensus has not been reached (median score 4–6 or where disagreement exists) as: ‘Include’ or ‘Exclude’ for consideration in the reporting guideline. Panellist will also be asked to score their confidence in their ratings on a 9-point Likert Scale (1, “not confident”, to 9, “very confident”).

#### Analysis

The demographic information will be summarised with descriptive statistics. The free text comments from round 1 and 2 will be coded and thematically analysed to identify the key issues and common themes. This information will inform the re-wording of items and the addition of new items consideration in subsequent rounds.

We will use a modified version of the RAND/UCLA appropriateness method to analyse the responses from each round. We modified this approach by asking panellists to rate “importance” rather than “appropriateness”. The RAND/UCLA appropriateness method considers the median panel rating and dispersion of each panel rating to provide an index of appropriateness/importance and agreement [24]. This involves calculating the median score, the IPR (30th and 70th) and the inter-percentile range adjusted for symmetry (IPRAS) for each item being rated. We will consider disagreement to be present in cases where IPR > IPRAS for a given item [24]. For the analysis of the round 1 and 2 responses, consensus for items to be considered for the reporting guideline will be categorised following the RAND/UCLA definitions [24]:

- “Include”: panel median of 7–9 for importance, without disagreement
- “Uncertain”: panel median of 4–6 for importance, or any median with disagreement
- “Exclude”: panel median of 1–3 for importance, without disagreement

For the analysis of round 3, consensus for items to be considered for the reporting guideline will be categorised as [27]:

- “Include”: panel majority as include
- “Exclude”: panel majority as exclude

#### Outcome of Delphi study

At the completion of this Delphi study, we will have reached consensus on a list of items that should be considered at the consensus meeting (stage 3). The report of the Delphi study will be submitted for publication.

### Stage 3 – consensus meeting

A face-to-face consensus meeting [28] will be held to decide on the most important reporting items to be included in the AGReMA statement and to develop the accompanying explanation and elaboration document [18]. The consensus meeting will follow the methods suggested for developers of health research reporting guidelines [18].

#### Procedure

The AGReMA working group will ensure that the collective expertise of participants reflect all relevant stakeholders (including trialists, epidemiologists, methodologists, statisticians, applied researchers and journal editors). Some key experts who participate in the Delphi study will be considered to participate in the consensus meeting. We will also invite experts who did not participate in the Delphi to achieve broad representation. Approximately 10 experts [28] will be invited to participate in a 1-day face-to-face consensus meeting. Prior to attending, the participants will be provided with the findings from the systematic review and the Delphi study. The meeting will involve presentations of the evidence for reporting quality of mediation studies, and results of the Delphi study. A member of the AGReMA working group will facilitate a structured discussion on the rationale of including each item identified in the Delphi study. Participants will be given opportunity to discuss each item. In cases of disagreement, an anonymised vote will be held to establish prioritisation of the item for inclusion in AGReMA. The meeting will conclude with discussion about the content and production of relevant documents (AGReMA statement, E&E paper, etc.) as well as strategies to optimise dissemination and implementation. Following the conclusion of the meeting, a written report on the meeting outcome will be circulated to the consensus meeting participants for comment and approval.

### Stage 4 development of the draft AGReMA statement and E&E document

The purpose of this stage is to draft the statement and accompanying E&E document to ensure that the wording and content is clear, precise, and suitable for all relevant stakeholders.

The purpose of the E&E document is to describe the background, rationale and justification for each reporting item and provide an example of clear reporting for each item. This is designed to help clarify the importance of each item, highlight relevant reporting issues, and assist authors in meeting the AGReMA statement requirements. The expert consensus meeting participants will be consulted to review and comment on the draft documents.

### Stage 5 – guideline dissemination

The goal of the final stage is to maximise the awareness, accessibility, and utilisation of AGReMA. The dissemination strategy will be informed and guided by the AGReMA working group and consensus meeting participants. We aim to produce simultaneous publications in several high-reach journals to begin the process of dissemination and uptake, accompanied by a social media dissemination strategy. We will liaise with relevant journal editors and funding agencies to encourage AGReMA endorsement alongside other reporting guidelines eg. CONSORT, STROBE etc. In addition to journal publications, we will make the AGReMA statement and its E&E document available on an open AGReMA web-domain, and index it on the Enhancing the QUAlity and Transparency Of health Research (EQUATOR) Network website and Penelope.ai [29]. We will create a suite of online resources including audio-visual guides which will be available on the AGReMA web-domain to assist application. The AGReMA working group will disseminate the statement at relevant conferences and statistical/methodological courses. Finally, the AGReMA statement and accompanying resources will be shared directly with authors that routinely use mediation analysis.

### Publication plan

- Publication 1: Study protocol
- Publication 2: Systematic review
- Publication 3: Delphi study
- Publication 4 & 5: simultaneous publications for the AGReMA statement and E&E paper

## Discussion

The number of studies using mediation analysis to understand the mechanisms of health exposures and interventions is increasing [1, 2]. However, the reporting of these studies remains heterogenous and incomplete [1, 5–11]. Methods for synthesising mediation studies are also under development so a reporting guideline is timely to help reduce reporting heterogeneity and facilitate the synthesis and pooling of mediation studies. A reporting guideline would not only facilitate proper reporting, but will also allow for accurate appraisal and implementation of the study findings by researchers, clinicians, patients, funders and policy makers.

The AGReMA project will produce a reporting guideline for studies that use mediation analysis to investigate causal mechanisms in healthcare research. To ensure this guideline is useful and widely used, it is being developed using comprehensive, robust and widely accepted methods [18]. We will also use a structured dissemination strategy to ensure implementation and uptake of AGReMA. We will ensure that the guideline is openly available and usable by accompanying it with a suite of resources to support its use.

## Data Availability

Not applicable

## Abbreviations

AGReMA: A guideline for reporting mediation analysis
CONSORT: Consolidated standards of reporting trials
E&E: Explanation and elaboration
EQUATOR: Enhancing the QUAlity and Transparency Of health Research
IPR: Inter-percentile range
IPRAS: Inter-percentile range adjusted for symmetry
MRC: Medical Research Council
NIH: National Institute of Health
NIHR: National Institute for Health Research
RAND/UCLA: Research ANd Development/University of California Los Angeles
REDCap: Research Electronic Data Capture
STROBE: Strengthening the Reporting of Observational Studies in Epidemiology
